# Supplementary material for: Molecular characterization of fowl adenovirus isolate of Malaysia attenuated in chicken embryo liver cells and its pathogenicity and immunogenicity in chickens
Source: PLoS One. 2019 Dec 31;14(12):e0225863. doi: 10.1371/journal.pone.0225863 (PMC6938336; doi:10.1371/journal.pone.0225863)
Supplement: S1 File — (DOCX) [file pone.0225863.s001.docx]

**Supporting Information S1 File**

[A] CELO : <https://www.ncbi.nlm.nih.gov/sites/myncbi/norfitriah.sohaimi.1/collections/59134329/public/>

[B] PL/060/08 : <https://www.ncbi.nlm.nih.gov/sites/myncbi/norfitriah.sohaimi.1/collections/59134333/public/>

[C] 340 : <https://www.ncbi.nlm.nih.gov/sites/myncbi/norfitriah.sohaimi.1/collections/59134340/public/>

[D] TR22 : <https://www.ncbi.nlm.nih.gov/sites/myncbi/norfitriah.sohaimi.1/collections/59134344/public/>

[E] KR5 : <https://www.ncbi.nlm.nih.gov/sites/myncbi/norfitriah.sohaimi.1/collections/59134346/public/>

[F] B1-7 : <https://www.ncbi.nlm.nih.gov/sites/myncbi/norfitriah.sohaimi.1/collections/59134352/public/>

[G] J2-A : <https://www.ncbi.nlm.nih.gov/sites/myncbi/norfitriah.sohaimi.1/collections/59134358/public/>

[H] C2B : <https://www.ncbi.nlm.nih.gov/sites/myncbi/norfitriah.sohaimi.1/collections/59134362/public/>

[I] SR48 : <https://www.ncbi.nlm.nih.gov/sites/myncbi/norfitriah.sohaimi.1/collections/59134366/public/>

[J] P7-A : <https://www.ncbi.nlm.nih.gov/sites/myncbi/norfitriah.sohaimi.1/collections/59134370/public/>

[K] SR49 : <https://www.ncbi.nlm.nih.gov/sites/myncbi/norfitriah.sohaimi.1/collections/59134375/public/>

[L] 75 : <https://www.ncbi.nlm.nih.gov/sites/myncbi/norfitriah.sohaimi.1/collections/59134382/public/>

[M] A-2A : <https://www.ncbi.nlm.nih.gov/sites/myncbi/norfitriah.sohaimi.1/collections/59134389/public/>

[N] 380 : <https://www.ncbi.nlm.nih.gov/sites/myncbi/norfitriah.sohaimi.1/collections/59134395/public/>

[O] 1047 : <https://www.ncbi.nlm.nih.gov/sites/myncbi/norfitriah.sohaimi.1/collections/59134398/public/>

[P] UF71 : <https://www.ncbi.nlm.nih.gov/sites/myncbi/norfitriah.sohaimi.1/collections/59134401/public/>

[Q] CR119 : <https://www.ncbi.nlm.nih.gov/sites/myncbi/norfitriah.sohaimi.1/collections/59134402/public/>

[R] YR36 : <https://www.ncbi.nlm.nih.gov/sites/myncbi/norfitriah.sohaimi.1/collections/59134407/public/>

[S] B-3A : <https://www.ncbi.nlm.nih.gov/sites/myncbi/norfitriah.sohaimi.1/collections/59134413/public/>

[T] 58 : <https://www.ncbi.nlm.nih.gov/sites/myncbi/norfitriah.sohaimi.1/collections/59134418/public/>

[U] TR59 : <https://www.ncbi.nlm.nih.gov/sites/myncbi/norfitriah.sohaimi.1/collections/59134421/public/>

[V] 764 : <https://www.ncbi.nlm.nih.gov/sites/myncbi/norfitriah.sohaimi.1/collections/59134431/public/>

[W] Australian FAdV Vaccine : <https://www.ncbi.nlm.nih.gov/sites/myncbi/norfitriah.sohaimi.1/collections/59134437/public/>

[X] 430-06 : <https://www.ncbi.nlm.nih.gov/sites/myncbi/norfitriah.sohaimi.1/collections/59134450/public/>

[Y] HG : <https://www.ncbi.nlm.nih.gov/sites/myncbi/norfitriah.sohaimi.1/collections/59134458/public/>

[Z] UPM08158 : <https://www.ncbi.nlm.nih.gov/sites/myncbi/norfitriah.sohaimi.1/collections/59134464/public/>

[AA] UPM08136 : <https://www.ncbi.nlm.nih.gov/sites/myncbi/norfitriah.sohaimi.1/collections/59134469/public/>

[BB] UPM04217 : <https://www.ncbi.nlm.nih.gov/sites/myncbi/norfitriah.sohaimi.1/collections/59134475/public/>

[CC] Duck Adenovirus : <https://www.ncbi.nlm.nih.gov/sites/myncbi/norfitriah.sohaimi.1/collections/59134476/public/>

[DD] Turkey Adenovirus : <https://www.ncbi.nlm.nih.gov/sites/myncbi/norfitriah.sohaimi.1/collections/59134479/public/>

[EE] CELO : <https://www.ncbi.nlm.nih.gov/sites/myncbi/norfitriah.sohaimi.1/collections/59134534/public/>

[FF] OTE : <https://www.ncbi.nlm.nih.gov/sites/myncbi/norfitriah.sohaimi.1/collections/59134537/public/>

[GG] PL/060/08 : <https://www.ncbi.nlm.nih.gov/sites/myncbi/norfitriah.sohaimi.1/collections/59134546/public/>

[HH] 08-3622 : <https://www.ncbi.nlm.nih.gov/sites/myncbi/norfitriah.sohaimi.1/collections/59134553/public/>

[II] 340 : <https://www.ncbi.nlm.nih.gov/sites/myncbi/norfitriah.sohaimi.1/collections/59134558/public/>

[JJ] Bareilly : <https://www.ncbi.nlm.nih.gov/sites/myncbi/norfitriah.sohaimi.1/collections/59134562/public/>

[KK] Kr-Yeoju : <https://www.ncbi.nlm.nih.gov/sites/myncbi/norfitriah.sohaimi.1/collections/59134570/public/>

[LL] Kr-Gunwi : <https://www.ncbi.nlm.nih.gov/sites/myncbi/norfitriah.sohaimi.1/collections/59134572/public/>

[MM] AG234 : <https://www.ncbi.nlm.nih.gov/sites/myncbi/norfitriah.sohaimi.1/collections/59134575/public/>

[NN] INT4 : <https://www.ncbi.nlm.nih.gov/sites/myncbi/norfitriah.sohaimi.1/collections/59134578/public/>

[OO] ON1 : <https://www.ncbi.nlm.nih.gov/sites/myncbi/norfitriah.sohaimi.1/collections/59134583/public/>

[PP] C2B : <https://www.ncbi.nlm.nih.gov/sites/myncbi/norfitriah.sohaimi.1/collections/59134595/public/>

[QQ] SR48 : <https://www.ncbi.nlm.nih.gov/sites/myncbi/norfitriah.sohaimi.1/collections/59134606/public/>

[RR] 685 : <https://www.ncbi.nlm.nih.gov/sites/myncbi/norfitriah.sohaimi.1/collections/59134615/public/>

[SS] SR49 : <https://www.ncbi.nlm.nih.gov/sites/myncbi/norfitriah.sohaimi.1/collections/59134622/public/>

[TT] 380 : <https://www.ncbi.nlm.nih.gov/sites/myncbi/norfitriah.sohaimi.1/collections/59136280/public/>

[UU] 05-50052-2924-1 : <https://www.ncbi.nlm.nih.gov/sites/myncbi/norfitriah.sohaimi.1/collections/59136283/public/>

[VV] 05-50052-3181 : <https://www.ncbi.nlm.nih.gov/sites/myncbi/norfitriah.sohaimi.1/collections/59136288/public/>

[WW] CR119 : <https://www.ncbi.nlm.nih.gov/sites/myncbi/norfitriah.sohaimi.1/collections/59136291/public/>

[XX] YR36 : <https://www.ncbi.nlm.nih.gov/sites/myncbi/norfitriah.sohaimi.1/collections/59136292/public/>

[YY] TR59 : <https://www.ncbi.nlm.nih.gov/sites/myncbi/norfitriah.sohaimi.1/collections/59136293/public/>

[ZZ] 764 : <https://www.ncbi.nlm.nih.gov/sites/myncbi/norfitriah.sohaimi.1/collections/59136303/public/>

[AAA] CFA3 : <https://www.ncbi.nlm.nih.gov/sites/myncbi/norfitriah.sohaimi.1/collections/59136295/public/>

[BBB] CFA40 : <https://www.ncbi.nlm.nih.gov/sites/myncbi/norfitriah.sohaimi.1/collections/59136299/public/>
