# Supplementary material for: Molecular characterization of fowl adenovirus isolate of Malaysia attenuated in chicken embryo liver cells and its pathogenicity and immunogenicity in chickens
Source: PLoS One. 2019 Dec 31;14(12):e0225863. doi: 10.1371/journal.pone.0225863 (PMC6938336; doi:10.1371/journal.pone.0225863)
Supplement: Attachment — Submitted filename: Response to Reviewers.docx [file pone.0225863.s002.docx]

**Response to Reviewers**

1) The manuscript title "MOLECULAR CHARACTERIZATION OF FOWL ADENOVIRUS ISOLATE OF MALAYSIA ATTENUATED IN CHICKEN EMBRYO LIVER CELLS AND IT’S PATHOGENICITY AND IMMUNOGENICITY IN SPECIFIC PATHOGEN FREE CHICKENS" is too long it may be changed and shortened?
see one example below.

"MOLECULAR CHARACTERIZATION OF A MALAYSIAN FOWL ADENOVIRUS ISOLATE ATTENUATED IN CHICKEN EMBRYO LIVER CELLS"

**Answer:** The study involved with attenuation of Fowl adenovirus in chicken embryo liver cells (CEL) and characterized molecularly the virus characteristic resulted from attenuation of virus in CEL. In addition to molecular characterization, the study also determine the pathogenicity and immunogenicity of the virus in chickens. Thus, the original title reflect more clearly on the study and contents of the manuscript. It is advisable to remove the specific pathogen free (SPF) to shorten the title. The CEL was prepared form liver of SPF chicken embryo as well as the pathogenicity and immunogenicity were conducted in SPF chickens. SPF was not mentioned in CEL. Thus, it justify to remove SPF for the chickens.

New title: "**MOLECULAR CHARACTERIZATION OF FOWL ADENOVIRUS ISOLATE OF MALAYSIA ATTENUATED IN CHICKEN EMBRYO LIVER CELLS AND IT’S PATHOGENICITY AND IMMUNOGENICITY IN CHICKENS**"

2) You should also describe about SPF chickens, either they were SPF broilers or layers?

**Answer:** The SPF chickens used in the study was from SPF White Leghorns layer chickens. It is included in the revised manuscript.
